# Hydrolysis of Ibuprofen Nitrile and Ibuprofen Amide and Deracemisation of Ibuprofen Using *Nocardia corallina* B-276 

**DOI:** 10.3390/molecules17033148

**Published:** 2012-03-12

**Authors:** Ricardo Lievano, Herminia Inés Pérez, Norberto Manjarrez, Aida Solís, Myrna Solís-Oba

**Affiliations:** 1 Departamento de Sistemas Biológicos, Universidad Autónoma Metropolitana Unidad Xochimilco, Calzada del Hueso No. 1100, Col. Villa Quietud, Delegación Coyoacán, C.P. 04960 México, D.F., Mexico; 2 Centro de Investigación en Biotecnología Ambiental, Instituto Politécnico Nacional, Carretera Estatal Santa Inés Tecuexcomac-Tepetitla Km 1.5, C.P. 90700, Tlaxcala, Mexico

**Keywords:** *Nocardia corallina*, deracemisation, ibuprofen, nitrile hydratase, amidase

## Abstract

A novel application of whole cells of *Nocardia corallina* B-276 for the deracemisation of ibuprofen is reported. This microorganism successfully hydrolysed ibuprofen nitrile to ibuprofen amide, and ibuprofen amide to ibuprofen, using a suspension of cells in a potassium phosphate buffer solution (0.1 M, pH = 7.0). These results can be explained by the presence of NHase and amidase enzymes, but the reactions are not enantioselective and low ee values were obtained. However, (*R*)-ibuprofen was isolated with >99% ee by a deracemisation process catalysed by *N.corallina* B-276. This is the first report of this kind of catalysis with this microorganism.

## 1. Introduction

Ibuprofen, (±)-2-(4-isobutylphenyl)propanoic acid, is one of the most well-known non-steroidal anti-inflammatory agents. Although (*S*)-ibuprofen is 100 times more active than its enantiomer, the racemate is still extensively used worldwide [1]. (*R*)-Ibuprofen undergoes metabolic chiral inversion to its enantiomer in the livers and kidneys of pigs and rats, but displays toxicity due to its storage as a hybrid glycerol ester in fatty tissue; the long-term effects of this are not known. Guidelines and regulations for the use of drugs strongly recommend the development of single enantiomers for new drugs. Therefore, a number of approaches to prepare optically pure drugs have been employed, which consider the resolution of diastereomeric salts, resolution of racemates, enzymatic kinetic resolution [2], and asymmetric synthesis, using chiral auxiliaries and chiral catalysts.

**Scheme 1:**
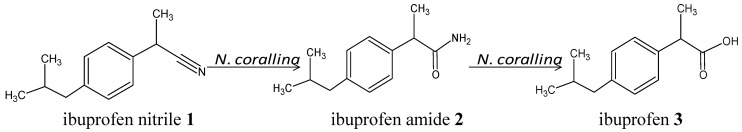
Biotransformation of ibuprofen nitrile **1** to ibuprofen amide **2** and ibuprofen **3** using *Nocardia corallina* B-276.

Recent studies have demonstrated enantioselective biotransformation of nitriles using nitrile-hydrolysing microorganisms [3,4]; we have also previously reported the ability of *Nocardia corallina* B-276 (a Gram-positive actinomycete) to hydrolyse achiral nitriles to amides [5]. It is noteworthy that the laboratory of Ohta, in 1991, was the first group to report the enzymatic hydrolysis of 2-substituted nitriles to give optically active 2-arylalkanoic acids [6]. Based on these reports, we were interested in evaluating the use of *N*. *corallina* to biocatalyse the enantioselective hydrolysis of racemic ibuprofen nitrile [2-(4-isobutylphenyl)propanenitrile, **1**], to optically active ibuprofen amide [2-(4-isobutyl-phenyl)propanamide, **2**] a prodrug [7,8], and to investigate whether this microorganism has the ability to hydrolyse amide **2** to optically active ibuprofen (**3**) (Scheme 1).

## 2. Results and Discussion

Two different methods were explored for performing the biotransformation of racemic **1** using *N. corallina* B-276 (Scheme 1): Method A, with the cells suspended in the culture media, in a 3-L bioreactor; and, Method B, with the harvested cells suspended in a phosphate buffer solution.

*Method A:* The biotransformation of **1**, in a 3-L bioreactor, was studied using three substrate:dry cells ratios 1:1.6, 1:8.3, and 1:12.1 to determine the influence of the quantity of cells on the extent of the reaction. The ratios were calculated by OD/dry cell weight, as described in the Experimental section. After 23 h, the amount of the unreacted nitrile **1** was high (63–78%) at all three nitrile:dry cell ratios; the increase in the ratio of cells from 1:1.6 to 1:12.1 did not significantly improve the hydrolysis. With regard to the hydrolysis products, the percentages of amide **2** were 11 and 19%, whereas the percentages of acid **3** were 11 and 18%, at ratios of 1:1.6 and 1:12.1, respectively. 

**Figure 1:**
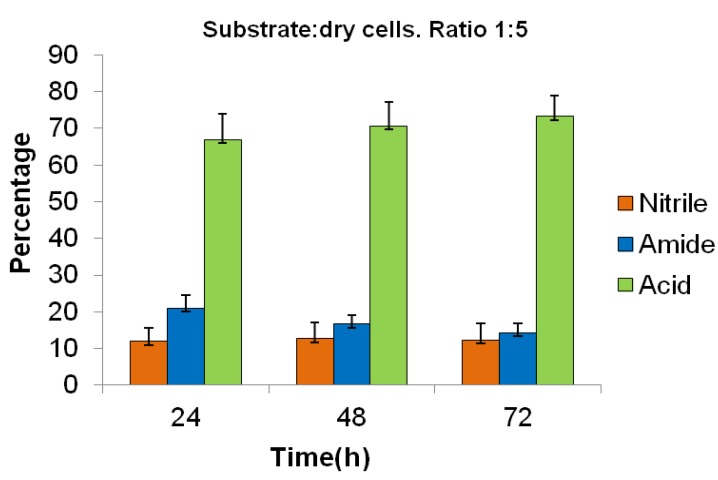
Biotransformation of ibuprofen nitrile **1**, using Method B, measured by GC. All the values were the mean of three independent experiments. Standard deviations for three independent experiments were represented by error bars.

*Method B:* The biotransformation of **1** using the harvested cells suspended in a phosphate buffer solution, with a substrate:dry cells ratio of 1:5, was significantly more efficient than the method using the bioreactor; and after 72 h, the percentages of unreacted **1** and of the products **2** and **3** were 12, 15, and 73%, respectively (Figure 1).

It is known that the biocatalysed hydrolysis of nitriles proceeds through two distinct mechanisms: (i) a direct nitrilase-catalysed biotransformation to the corresponding carboxylic acid [8], or (ii) a 2-step process, involving first a nitrile hydratase (NHase) followed by the hydrolysis of the amide to the carboxylic acid by an amidase [9]. In the biotransformation of **1** catalysed by *N. corallina,* both the amide (**2**) and the acid (**3**) were isolated in 15% and 73% yields respectively, which strongly suggests that hydrolysis of ibuprofen nitrile using *N. corallina* proceeded according to the second mechanism, and implies that NHase and amidase enzymes were present. Moreover, the known ability of nocardiaform actinomycetes, especially of the genus *Rhodococcus* [10], to hydrolyse organic nitriles via nitrile hydratase/amidase, and the proposal by Layh *et al.* [11] that Gram-negative strains predominantly hydrolyse nitriles via nitrilases, while in Gram-positive strains nitrile hydratase/amidase systems dominate, give additional support to the suggestion that hydrolysis of **1** by *N. corallina* follows the second mechanism.

The maximum yield of each enantiomer from the enantioselective hydrolysis of a racemic compound should be 50% with a 100% ee. The NHase from *N. corallina *was not enantioselective towards the hydrolysis of the racemic ibuprofen nitrile **1**, because the percentage of unreacted **1** was 12% (Figure 2). The hydrolysis of the resulting amide **2**, catalysed by the amidase from *N. corallina*, was not enantioselective either, as, after 72 h, 15% of **2** remained, with an enantiomeric ratio of 69/31 (ee 38%). However, the hydrolysis of **2** to *R*-ibuprofen proceeded in 73% yield with an ee >99%, as determined by chiral HPLC. It is evident that the NHase and the amidase are not enantioselective enough to explain this result, as **3** would otherwise have been obtained with a lower optical purity. Hence, another biotransformation seems to occur in the **2→3** step, which could be explained by a deracemisation process involving the stereoinversion of *S*-**3** to *R*-**3**. Mitsukura *et al. *[12], reported the deracemisation of *rac*-phenylpropionic acid to the *R*-enantiomer using *Nocardia diaphanozonaria* through an isomerase-involving reaction. However, when Kato *et al. *[13] explored the biotransformation of two well-known racemic NSAIDs (*i.e.*, ibuprofen and flurbiprofen) with this microorganism, they found a very low deracemisation activity (9 and 6% ee, respectively), in striking contrast with the 99% ee we obtained for *rac*-ibuprofen using *N. corallina *B-276. 

Next, we studied the effect of the biocatalyst ratio on the biotransformation of the nitrile **1, **to the amide **2**, and the biocatalysed hydrolysis of this compound to ibuprofen **3**, using Method B. When the substrate:dry cells ratio was increased to 1:17, only 59% of **1** had been hydrolysed within 24 h and low percentages of products **2** and **3** (39 and 20%, respectively) were observed. The enantiomeric ratios were 49/51, 65/35, and 54/46 (*R*/*S*), respectively, indicating that the hydrolysis was not enantioselective. After 48 h, the percentages were 19% of **1**, 52% of **2**, and 29% of **3**; however, the ee of **3** was improved to 78%. An increase in the proportion of cells did not improve the yield, but rather reduced the conversion. From these results, it is evident that the NHase is more active than the amidase. More notable was the deracemisation process in this experiment: after 24 h, **3** was almost racemic, whereas after 48 h, the ee of the *R*-acid had increased to 78%.

We then explored the amidase activity of this microorganism on the amide **2**, using Method B. We found that the amidase activity was low with a 1:17 substrate:dry cells ratio and resulted in only 34% conversion after 96 h; the residual **2** had an enantiomeric ratio of 61/39 (*ee* 22%) while the *ee* of *R*-ibuprofen was >99%. Kato *et al.* [13] reported that 2-phenylpropanamide was not recognised by either the amidase or the deracemisation enzymes from *N. diaphanozonaria,* and the amide was recovered unchanged after 48 h (97% recovery). In contrast, *N**.** corallina* could use amide **2** as substrate to generate (*R*)-ibuprofen with a high ee.

To demonstrate that *N. corallina* was able to perform the stereoinversion of the chiral centre, we studied the biotransformation of commercial *S*-ibuprofen (ee >99%) with *N. corallina*. After a 72 h treatment using Method B (substrate: dry cells-ratio of 1:5), the enantiomeric ratio was 71:29 as determined by HPLC; the major enantiomer being (*R*)-ibuprofen. This result confirmed the novel activity we found for this microorganism to generate (*R*)-profens. 

It has been proposed that the mechanism of stereoinversion occurs via the formation of an ‘activated’ acyl-CoA-derivative of the (*S*)-acid, followed by epimerisation to the (*R*)-isomer and hydrolysis of the (*R*)-acyl-CoA-ester [13,14].

## 3. Experimental

### 3.1. General

*rac*-Ibuprofen and (*S*)-ibuprofen were purchased from Aldrich. Ibuprofen nitrile and ibuprofen amide were synthesised as described by Yamamoto *et al. *[15]. The cells of *N. corallina* B-276 (ATCC 31338) were grown following the method reported by Pérez *et al. *[5]. The biocatalysed hydrolysis of **1** was carried out according to our previously reported conditions [5,16,17]: 

*Method A*: Biotransformation in a 3-L bioreactor, using the following conditions: agitation rate 226 rpm, aeration rate 0.9 vvm (air only), pH 8.4, and temperature 28–30 °C. To determine the correlation between optical density and dry cell weight, samples were withdrawn every hour, the optical density (OD_660 nm_) was measured, and the cells were dried and weighed to determine the concentration of dry cells (g·L^−1^) in the culture. Using these data, a growth curve was plotted; the highest concentration of biomass was achieved in 6 h [17]. Using OD measurement and the previously reported growth curve of *N. corallina* [17], we estimated three substrate:dry cells ratios at the outset of this study, viz., 1:1.6, 1:8.3, and 1:12.1 (w/w), in 1,800 mL of culture media. As an example of the experiment, at a 1:8.3 ratio, ibuprofen nitrile (0.627 g, 3.35 mM) in *N,N*-dimethylformamide (13.2 mL) was added, and the mixture was incubated for 24, 46, 72, and 96 h.

*Method B*: The cells were harvested [5] and suspended in a phosphate buffer solution (0.1 M, pH 7.0). Two substrate:dry cells ratios were used in the biotransformation: 1:5 and 1:17 (w/w). In this experiment, ibuprofen nitrile (0.02 g, 0.107 mM) in *N,N*-dimethylformamide (0.3 mL) was added to the cell suspension and the reaction mixture (50 mL), was incubated for 24, 48, and 72 h. The biotransformation of ibuprofen amide was performed using Method B with a substrate:dry cells ratio (w/w) of 1:17. Ibuprofen amide (0.037 g, 0.18 mM) in 0.6% (v/v) of *N,N*-dimethylformamide was added to the cell suspension and the mixture was incubated for 96 h in 50 mL of a phosphate buffer (0.1 M, pH 7.0). All experiments were repeated in triplicate.

The extent of conversion was determined by GC analysis on a Hewlett-Packard HP 6890 gas chromatograph equipped with a flame ionisation detector and an HP-5 column (30 m × 0.33 mm) at 180 °C, with N_2_ as the carrier gas, at a flow rate of 1.0 mL·min^−1^.

The enantiomeric excess (ee) was measured by chiral HPLC analyses performed on an Agilent 1100 liquid chromatograph equipped with a diode array detector and a Chiralcel OD (L × ID: 25.0 × 0.46 cm) column. The chromatographic conditions were as follows: (a) For ibuprofen nitrile, **1**, the mobile phase was hexane isopropanol (99.5:0.5 with 0.1% TFA) at a flow rate of 0.8 mL·min^−1^ at 25 °C; recorded at 220 nm. (b) For ibuprofen amide, **2**, the mobile phase was hexane-isopropanol (90:10) at a flow rate of 0.8 mL·min^−1^ at 25 °C; recorded at 220 nm. The retention times for each enantiomer were 9.58 and 10.45 min respectively. (c) For ibuprofen (**3)**, a Chiralcel OJ-H (L × ID: 25.0 × 0.46 cm) column was used with a hexane-isopropanol (95:5) mobile phase at a flow rate of 0.6 mL·min^−1^ at 25 °C, recorded at 220 nm. The retention times were 9.18 and 9.87 min for the (*R*)-and (*S*)-enantiomers, respectively, and were assigned by comparing the obtained values with the retention time of commercial (*S*)-ibuprofen under the chromatographic conditions described for *rac*-ibuprofen or the product, **3**.

The products were identified by IR and ^1^H-NMR and ^13^C-NMR spectroscopy. The IR spectra were recorded on a Perkin-Elmer Paragon 1600 FT with the samples as liquid films, while the ^1^H-NMR and ^13^C-NMR spectra were recorded on a Varian 400 MHz instrument using CDCl_3_ as the solvent and TMS as the internal reference.

### 3.2. Physical and Spectroscopic Data

*(±)-2-(4-Isobutylphenyl)propanenitrile *(*ibuprofen nitrile*, **1**): IR (film) ν: 2240 cm^−1^; ^1^H-NMR: δ = 7.26 (d, *J* = 8.4 Hz, 2H), 7.15 (d, *J* = 8.4 Hz, 2H), 3.87 (q, *J* = 7.2 Hz, 1H), 2.46 (d, *J* = 7.6 Hz, 2H), 1.85 (m, 1H), 1.63 (d, *J* = 7.2 Hz, 3H), 0.90 ppm (d, *J* = 6.4 Hz, 6H); ^13^C-NMR: δ = 141.6, 134.2, 129.8, 126.4, 121.8, 44.9, 30.9, 30.2, 22.3, and 21.4 ppm.

*(±)-2-(4-Isobutylphenyl)propanamide *(*ibuprofen amide*, **2**): IR (film) ν: 3349.8, 3175.1, and 1656.7, 1641.4 cm^−1^; ^1^H-NMR: δ = 7.20 (d, *J* = 8.0 Hz, 2H), 7.12 (d, *J* = 8.0 Hz, 2H), 5.41 and 5.30 (b, 2H, NH_2_), 3.58 (q, *J* = 7.2 Hz, 1H), 2.46 (d, *J* = 7.6 Hz, 2H), 1.85 (m, 1H), 1.52 (d, *J* = 7.2 Hz, 3H), 0.90 (d, *J* = 6.8 Hz, 6H); ^13^C-NMR: δ = 176.4, 140.6, 129.4, 126.9, 46.1, 44.8, 30.1, 22.3, and 18.2 ppm; m.p. = 112–114 °C [18]. 

*2-(4-Isobutylphenyl)propanoic acid *(*ibuprofen*, **3**): IR (film) ν: 3090, 3025, 2956, and 1740, 1707 cm^−1^; ^1^H-NMR: δ = 7.23 (d, *J* = 8.0 Hz, 2H), 7.13 (d, *J* = 8.0 Hz, 2H), 3.71 (q, *J* = 7.2 Hz, 1H), 2.48 (d, *J* = 7.2 Hz, 2H), 1.86 (m, 1H), 1.52 (d, *J* = 7.2 Hz, 3H), 0.93 ppm (d, *J* = 6.8 Hz, 6H); ^13^C-NMR: δ = 180.8, 140.8, 136.9, 129.4, 127.3, 45.22, 45.14, 30.4, 22.7, and 18.4 ppm; m.p. = 49–50 °C. 

## 4. Conclusions

*N. corallina* B-276 showed nitrile hydratase and amidase activities, but stereoselectivities of these enzymes were low. However, it was found that *N. corallina* catalyses a deracemisation process to biotransform almost racemic ibuprofen, which was obtained from the hydrolysis of the ibuprofen amide, to the (*R*)*-*ibuprofen enantiomer with ee >99%*. *This indicates that *N. corallina *B-276 is a novel biocatalyst that is capable of performing deracemisation processes. Recent pharmacological studies have focused attention on the (*R*)-enantiomer of this type of non-steroidal anti-inflammatory agent [19]; for this reason, recent efforts have been directed at resolving *rac*-ibuprofen (**3**) [20]. We are currently investigating further evidence and examples of this novel inversion, including the application of this methodology to other α-arylpropionic acid derivatives. This microbial transformation might form the basis of a biocatalytic process for the production of optically pure (*R*)-profens.
